# Seizure suppression through manipulating splicing of a voltage-gated sodium channel

**DOI:** 10.1093/brain/awv012

**Published:** 2015-02-12

**Authors:** Wei-Hsiang Lin, Miaomiao He, Richard A. Baines

**Affiliations:** Faculty of Life Sciences, University of Manchester, Manchester, UK

**Keywords:** *Drosophila*, voltage-gated sodium channel, splicing, seizure, hyperexcitability

## Abstract

Seizure can result from increased voltage-gated persistent sodium current expression. Although many clinically-approved antiepileptic drugs target voltage-gated persistent sodium current, none exclusively repress this current without also adversely affecting the transient voltage-gated sodium current. Achieving a more selective block has significant potential for the treatment of epilepsy. Recent studies show that voltage-gated persistent sodium current amplitude is regulated by alternative splicing offering the possibility of a novel route for seizure control. In this study we identify 291 splicing regulators that, on knockdown, alter splicing of the *Drosophila* voltage-gated sodium channel to favour inclusion of exon K, rather than the mutually exclusive exon L. This change is associated with both a significant reduction in voltage-gated persistent sodium current, without change to transient voltage-gated sodium current, and to rescue of seizure in this model insect. RNA interference mediated knock-down, in two different seizure mutants, shows that 95 of these regulators are sufficient to significantly reduce seizure duration. Moreover, most suppress seizure activity in both mutants, indicative that they are part of well conserved pathways and likely, therefore, to be optimal candidates to take forward to mammalian studies. We provide proof-of-principle for such studies by showing that inhibition of a selection of regulators, using small molecule inhibitors, is similarly effective to reduce seizure. Splicing of the *Drosophila* sodium channel shows many similarities to its mammalian counterparts, including altering the amplitude of voltage-gated persistent sodium current. Our study provides the impetus to investigate whether manipulation of splicing of mammalian voltage-gated sodium channels may be exploitable to provide effective seizure control.

## Abstract (precis)

Voltage-gated persistent sodium current (INaP) is a tractable target for antiepileptic drugs. Using a strategy focused on INaP reduction, Lin *et al.* identify 95 regulators of voltage-gated sodium channel splicing for which RNAi knockdown reduces seizure duration in *Drosophila*. Manipulation of splicing regulators could improve control of epilepsy.

## Introduction

Although mutations in more than 60 genes have been linked to epilepsy (Noebels, 2003), a principle commonality underlying seizure generation is neuronal hyperexcitability. Multiple lines of evidence implicate an abnormal increase in neuronal voltage-gated persistent sodium current (I_NaP_) directly contributes to hyperexcitability and, as such, this current component is an attractive target for antiepileptic drug (AED) design (Chen *et al.*, 2001; Stafstrom, 2007). However, to date, no clinically approved AEDs are available to selectively target I_NaP_ without also impacting transient voltage-gated sodium current (I_NaT_), which is critical for normal action potential firing.

It is well established that voltage-gated sodium (Na_v_) channels rapidly inactivate after brief openings following depolarization of the neuronal membrane. What is less well understood is the mechanism through which inactivated channels briefly reopen to mediate I_NaP_ (Chen *et al.*, 2001; Stafstrom, 2007). Regardless of these uncertainties, it is known that although I_NaP_ carries considerably less current than I_NaT_, its presence can have a profound influence on membrane excitability as it is able to keep a neuronal membrane depolarized for long periods of time (Yue *et al.*, 2005). Indeed, the relative potency of clinically used AEDs such as phenytoin, valproate and lamotrigine almost certainly derive from their ability to potently reduce this conductance, in addition to inhibiting I_NaT_ (Chao and Alzheimer, 1995; Taverna *et al.*, 1998; Spadoni *et al.*, 2002).

Understanding the molecular machinery that regulates I_NaP_ is poor, which is partly because of the relative complexity generated by the presence of multiple sodium channel genes (*SCN1A–SCN11A*) in the mammalian genome, all of which show differing levels of this current (Goldin, 2001; Lin and Baines, 2015). Several lines of evidence suggest that I_NaP_ amplitude can be regulated by mRNA alternative splicing. For example, splicing at exon 5 in human *SCN1A* is mutually exclusive with the choice of either exons 5A or 5N (for adult and neonatal). Heterologous expression of human *SCN1A*-5N, in HEK293T cells, produces channels that exhibit more rapid inactivation and reduced I_NaP_ compared to *SCN1A*-5A (Fletcher *et al.*, 2011). Alternative splicing in this region (exon 5 or 6) is also observed in *SCN2A*, *SCN3A*, *SCN8A* and *SCN9A* in both humans and mice (Sarao *et al.*, 1991; Yarowsky *et al.*, 1991; Gustafson *et al.*, 1993; Kasai *et al.*, 2001; Raymond *et al.*, 2004). Intriguingly, the observed increased inclusion of exon 6N in both *Scn2a* and *Scn3a* following electrical or kainite-induced seizure in adult rat hippocampus implies a correlation between splicing and seizure generation (Gastaldi *et al.*, 1997; Aronica *et al.*, 2001). A somewhat clearer picture of how splicing affects I_NaP_ has emerged from studies in *Drosophila melanogaster* (Lin *et al.*, 2009, 2012). In contrast to mammals, insects contain only one Na_v_ channel homologue, encoded by *paralytic* (*DmNa_v_*, currently known as *para*) (Feng *et al.*, 1995). Splicing at exon 25 in *DmNa_v_* mirrors that observed at exon 5 in *SCN1A*: one of a pair of mutually-exclusive exons (termed K and L in the fly) encodes region S3–4, which contributes to the voltage sensor. Channels containing exon L exhibit significantly larger I_NaP_ compared to those containing exon K, with no change in I_NaT_ (Lin *et al.*, 2009). Increased inclusion of exon L, along with an enlarged I_NaP_ in motorneurons, is characteristic of bang-sensitive mutants (e.g. *sda* and *eas*) that exhibit lower seizure threshold and increased seizure duration in response to electric shock (Lin *et al.*, 2012). Splicing of exon 25 is, moreover, activity-dependent with activity increasing inclusion of exon L, which in turn increases action potential firing leading to a reinforcing positive feedback. Manipulating splicing to increase exon K expression uncouples this feedback cycle, reduces I_NaP_ and rescues seizure-like behaviour in these same seizure mutants (Lin *et al.*, 2012).

Splicing at exon 25 is modified by pasilla, a K homology (KH) domain-containing RNA binding protein (Park *et al.*, 2004; Lin *et al.*, 2012). Knockdown of pasilla expression increases inclusion of exon K, decreases I_NaP_ and, importantly, provides effective rescue of seizure (Lin *et al.*, 2012). Thus, understanding the regulatory mechanisms that orchestrate splicing in *Na_v_* transcripts may be exploitable for the design of AEDs that have high specificity for targeting I_NaP_. The mammalian homologues of pasilla, NOVA1 and NOVA2, also regulate *SCN* alternative splicing (Ule *et al.*, 2003, 2006). Like pasilla, NOVA recognizes YCAY motifs located in introns (which flank both exon 5/6 in mammalian *SCNs* and exon 25 in *DmNa_v_*). Moreover, a number of observations link NOVA function with epilepsy. Mesial temporal lobe epilepsy has been associated with an upregulation of *NOVA2* and *SCN1A-5N* transcript abundance (Heinzen *et al.*, 2007). Perturbation of NOVA steady-state levels in *Nova2^+/−^* heterozygous mice gives rise to cortical hyperexcitability and to spontaneous generalized seizure discharge (Eom *et al.*, 2013). NOVA localization shifts from primarily nuclear to cytoplasmic within hours after pilocarpine-induced seizure (Eom *et al.*, 2013). These, and additional, observations highlight an important and perhaps exploitable relationship between *SCN* mRNA splicing, NOVA and epilepsy. The conservation of function between pasilla and NOVA offers the opportunity to use the tractability of *Drosophila* to rapidly identify underlying signalling pathways.

In this study, we generated luciferase-based mini-genes to report splicing at exon 25 in *DmNa_v_*. Expression in S2R+ cells and exposure to a *Drosophila* double-stranded RNA library identified 291 genes that, on knockdown, increased inclusion of exon K (sufficient to reduce I_NaP_). Expression of RNA interference (RNAi) *in vivo* shows that knockdown of 95 of these genes provides significant behavioural rescue of induced-seizure in two bang-sensitive mutants. We further show that small molecule inhibitors of the protein products of some of the targeted genes are effective anticonvulsants.

## Materials and methods

### Mini-gene construction

Genomic DNA was extracted in 50 µl extraction buffer (10 mM Tris-HCl, 1 mM EDTA, 25 mM NaCl and 200 µg/ml proteinase K) and incubated at 37°C for 30 min. *DmNa_v_* genomic DNA, spanning exon 24 to exon 26, was amplified by PCR (Phusion® High-Fidelity DNA Polymerase, New England Biolabs) that consisted of the following in a total volume of 50 µl: 20 pmol primers, dNTPs at 0.2 mM each, and 1× Phusion HF buffer with 1.5 mM Mg^2+^. Forward primer (5′-gatctggtacc**ATG**GCATTAGAAGATGTACATCTGCCAC-3′), located at exon 24, introduced a *Kpn*I site and a translational initiation codon. Reverse primer (5′-gttatgcggccgctctagaCTTAAAATATTTTCCAGCAAAAAGCTG-3′), located at exon 26, introduced an *Xba*I and *Not*I sites. Cycling conditions were: initial denaturation at 98°C for 5 min; 35 cycles of 98°C for 10 s, 55°C for 20 s and 72° for 4 min; a final extension step at 72°C for 10 min. The PCR product was digested with *Kpn*I and *Not*I and ligated into pBluescript® II KS vector (Stratagene Inc). A Luciferase reporter gene, *renilla* or *firefly*, was inserted in-frame to the 3′ end of exon 26. Both *renilla* and *firefly* genes were PCR amplified and *Xba*I and *Not*I sites introduced at the 5′ and 3′ ends, respectively. The primer pairs (5′ to 3′) are: *renilla*, gtacatctagaATGACTTCGAAAGTTTATGATCCAGAA and gttatgcggccgcTTATTGTTCATTTTTGAGAACTCGCTC; *firefly*, gtacatctagaATGGAAGACGCCAAAAACATAAAGA and gttatgcggccgcTTACACGGCGATCTTTCCGCC. To report K exon expression, (*K-renilla* mini-gene) a termination codon was inserted in exon L by site-directed mutagenesis. In the same way, a termination codon was introduced in exon K in the *L-firefly* mini-gene. *K-renilla* or *L-firefly* mini-genes were then digested with *Kpn*I and *Not*I and ligated into a pAc5.1 vector (Invitrogen). All clones were checked by sequencing prior to expression analysis.

### Genome-wide double-stranded RNA library screen

S2R+ cells (1.5 × 10^4^ cells in 15 µl of Insect Express Prime media, PAA) were treated with 250 ng of double-stranded RNA (∼21 000 double-stranded RNAs, ∼98.8% coverage, covering ∼14 000 protein encoding genes and ∼1000 non-coding genes on 53 × 384 well plates) for 48 h and followed by co-transfection (Effectene®, QIAGEN) of *K-renilla* and *L-firefly* mini-genes (10 ng each) for a further 48 h. The transfection procedure is as described in the manufacturer’s instructions (QIAGEN). S2R+ cells were lysed with 0.35% Triton™ X-100 in BL buffer (50 mM HEPES, 0.5 mM EDTA, 0.36 mM phenylacetic acid and 0.07 mM oxalic acid) and coelenterazine-h (3 μM, Promega) added to measure K-renilla luciferase activity. Renilla-luciferase activity declined completely after 10 min and d-Luciferin (0.46 mM, Molecular Probes) was then added to measure L-firefly luciferase activity. A Varioskan® flash plate reader (Thermo Scientific) was used to measure luminescence.

### RNA extraction and reverse transcription

Total RNA was extracted from 30 male adult heads using the RNeasy® micro kit (QIAGEN). cDNA synthesis was carried out in 20 μl total volume. Oligo(dT) (0.5 µg) and random hexamers (0.2 µg) were mixed with RNA and made up to 12 µl with RNase-free water. The mix was incubated at 65°C for 5 min to denature RNA followed by incubation on ice for 2 min. To this was added 4 µl of reaction buffer (in mM: 250 Tris-HCl, 250 KCl, 20 MgCl_2_, 50 DTT), 2 µl of 10 mM dNTPs, 1 µl of RNase inhibitor and 1 µl of RevertAid™ M-MuLV (monkey murine leukaemia virus) reverse transcriptase (RevertAid™ First Strand cDNA Synthesis kit, Fermentas). The reaction was incubated at 25°C for 10 min, 42°C for 60 min followed by 70°C for 10 min.

### Determination of exon inclusion

The determination of ratio of exon K to exon L inclusion in *DmNa_v_* from whole CNS is described in Lin *et al.* (2012).

### Quantitative PCR

Quantitative PCR was performed using SYBR Green I real-time PCR method (Roche, LightCycler® 480 SYBR® Green I Master). The Ct values, as defined by the default setting, were measured using a LightCycler® 480 II realtime PCR (Roche) using a thermal profile of 10 min at 95°C followed by 45 cycles of 10 s at 95°C, 10 s at 60°C and 10 s at 72°C. Single-product amplification was confirmed by post-reaction dissociation analysis. PCR primers were designed with the aid of LightCycler® Probe Design Software 2.0 (v1.0) (Roche). Primer sequences (5’ to 3’) are listed in Supplementary Table 1. Relative gene expression was calculated as the 2^−ΔCt^, where ΔCt was determined by subtracting the average *Rp49* Ct value from that for each gene.

### Fly stocks

Flies were maintained on standard cornmeal medium at 25°C. *Bas^1^* and *bss^1^* were gifts from Dr Kevin O’Dell (University of Glasgow). Wild-type was Canton-S. The UAS-RNAi *pasilla* (stock no. 33 426) was obtained from Bloomington and all other UAS-RNAi lines (Supplementary Table 2) were obtained from the Vienna *Drosophila* Resource Centre. *Bas^1^*;Gal4*^Cha^* and *bss^1^*;Gal4*^Cha^* were derived by crossing *bas^1^* (*bang sensitive^1^*) or *bss^1^* (*bang sensless^1^*) with *Cha^B19^*-Gal4 (gift from Dr Paul Salvaterra, City of Hope, USA).

### Behavioural screening on bang sensitive mutants

Twenty virgin females of *bas^1^*;Gal4*^Cha^* were crossed with five UAS-RNAi males. Because *bas^1^* is on the X chromosome and heterozygous *bas^1^*/*+* females show significantly reduced mean recovery time (28.3 ± 4.3 s), we used *bas^1^*/*Y* hemizygous males (232.7 ± 26.2 s) for the behavioural screening. Flies (2–3 days old) were tested at least 1 day after collection to ensure total recovery from CO_2_-anaesthesia. Flies were transferred to an empty vial and left to recover for 30 min before mechanical shock by vortexing the vial at maximum speed for 10 s. Mean recovery time was calculated from the average time taken for all 10 flies to recover from paralysis to standing. At least three replicates were performed for each RNAi line. Values were compared to control flies (*bas^1^/Y*;Gal4*^Cha^/+*) by ANOVA with Tukey’s post-test. Results were deemed significant at either *P ≤ 0.05 or **P ≤ 0.01. In the same way, we cross virgin females of *bss^1^*;Gal4*^Cha^* with UAS-RNAi males and the F1 male flies (*bss^1^/Y*;Gal4*^Cha^/*UAS-RNAi) were tested.

### Acute exposure of chemical inhibitors

Groups of 10 young adult male flies (*bas^1^*/*Y*) within 8 h of eclosion were placed in an empty vial containing filter paper soaked with sucrose (5%) and drug. Flies were kept in the vial for 24 h at which point the filter paper was removed. Flies were left to recover for 30 min before being vortexed. Mean recovery times and statistical significance were determined as described above. The chemical inhibitors and the solvent used were: phenytoin (D4505, Sigma) dissolved in H_2_O/0.1 N NaOH solution (3:1); dipyridamole (D9766, Sigma) and rapamycin (10798668, Fisher Scientific) dissolved in ethanol; etoposide (E1383, Sigma) dissolved in ethanol/DMSO solution (5:1); isethionate (PZ0199, Sigma) and antipain dihydrochloride (A6191, Sigma) dissolved in H_2_O. These solvents were also fed to the respective control (*bas^1^*/*Y*) flies and did not show significant effect to mean recovery time.

### Electrophysiology

Methods used to identify anterior corner cell motorneurons and isolate and record sodium currents are described in Marley and Baines (2011).

## Results

### Mini-gene reporters for splicing of exons K and L

**Figure 1:**
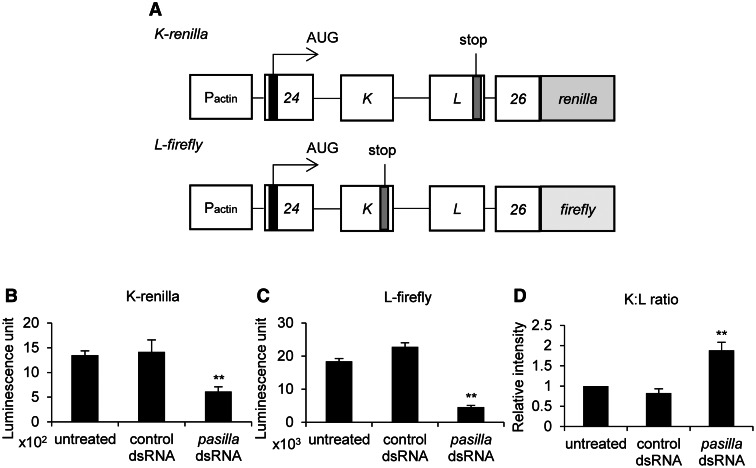
**Luciferase-based mini-genes report splicing of exons K and L in *DmNa_v_***. (**A**) Mini-gene cassettes driven by actin promoters (Pactin), contain *DmNa_v_* genomic DNA spanning exon 24 to exon 26 connected in-frame to either a *renilla* or a *firefly* luciferase reporter gene. A translational initiation codon is introduced in exon 24. A termination codon is introduced in exon L of the *K-renilla* and in exon K of *L-firefly*. (**B** and **C**) Double-stranded RNA-mediated knockdown of *pasilla*, a *DmNa_v_* exon K/L splicing regulator, suppressed mini-gene expression of (**B**) *K-renilla* (1346 ± 91 versus 1415 ± 244 versus 614 ± 93 luminescence units, untreated versus control double-stranded RNA versus *pasilla* double-stranded RNA, respectively) and (**C**) *L-firefly* (18 × 10^3 ^± 858 versus 23 × 10^3 ^± 1274 versus 4552 ± 548 units, untreated versus control double-stranded RNA versus *pasilla* double-stranded RNA, respectively) in S2R+ cells. (**D**) *pasilla* double-stranded RNA altered the K:L ratio to favour increased inclusion of K (K-renilla:L-firefly ratio 1.9 ± 0.2) compared to untreated (which was set at 1) or control double-stranded RNA treated cells (0.8 ± 0.1). Control double-stranded RNA used is BKN21565 (CG11360), known to regulate splicing of *DmNa_v_* exons 11 and 12 but not exon 25 (Park *et al.*, 2004). Values (*n* = 3, mean ± SEM) were compared by a Student’s *t*-test and results were deemed significant at **P ≤ 0.01. dsRNA = double-stranded RNA.

To identify regulators of splicing at exon 25 (i.e. exons K or L) in *DmNa_v_*, we constructed two mini-gene reporters (Fig. 1A). Each reporter, driven by an actin promoter, contains *DmNa_v_* genomic DNA spanning exon 24 to exon 26 connected in-frame to a luciferase reporter gene (*renilla* or *firefly*) and a translational initiation codon artificially introduced in exon 24. In *K-renilla*, a termination codon was introduced in exon L, such that inclusion of exon K leads to expression of a mRNA encoding a *renilla*-fusion protein, while inclusion of exon L results in a truncated, and non-functional, transcript. In the same way, a termination codon was introduced in exon K in *L-firefly*, such that inclusion of exon L expresses a mRNA encoding a *firefly*-fusion protein, while inclusion of exon K results in a truncated protein. The ratio between *renilla:firefly* luciferase activities effectively reports the K:L ratio.

To determine functionality of the mini-gene cassettes, we transfected them into S2R+ cells and confirmed both *renilla* and *firefly* luciferase activity (Fig. 1B and C). Knockdown of pasilla predictably altered the K:L ratio to favour increased inclusion of K (K:L 1.9 ± 0.2) compared to untreated (which was set at 1) or control double-stranded RNA treated cells (0.8 ± 0.1) (Fig. 1B–D). RNAi-mediated knockdown of pasilla also results in reduced expression of both *renilla* and *firefly* luciferase reporters to 46% and 25% (*n* = 5, *P* ≤ 0.01), respectively, compared with untreated cells (Fig. 1B and C). Indeed, this was a common effect noted with many of the double-stranded RNAs that we tested (the reduction is quantified in Supplementary Table 2). Regardless of effect to expression level, our results confirm that S2R+ cells have the required machinery to splice exons K and L in *DmNa_v_* and that the mini-genes effectively report this splicing event.

### A genome-wide RNAi screen to identify regulators of splicing

**Figure 2:**
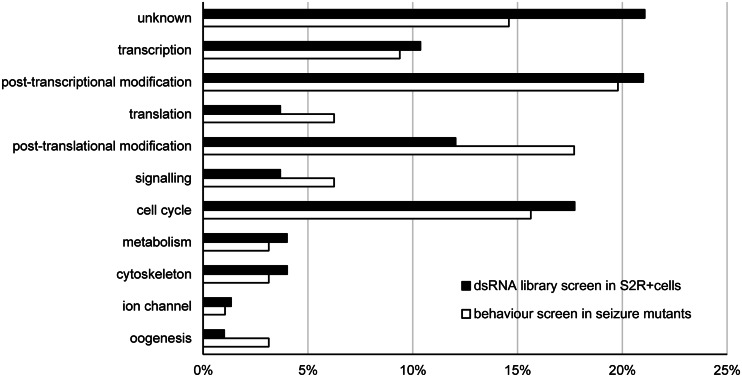
***DmNav* splicing regulators**. Two hundred and ninety-nine double-stranded RNAs (targeting 291 genes, ∼1.4% of the genome) increased inclusion of exon K in *DmNa_v_* (black bar). Ninety-five of these corresponding UAS-RNAi lines rescue seizure duration in both seizure mutants, *bang sensitive^1^* and *bang senseless^1^* (white bar). These genes can be classified into 11 categories according to Gene Ontology annotation (Boyle *et al.*, 2004; Camon *et al.*, 2004). The percentages with categories are indicated. See Supplementary Table 2 for details of these genes. dsRNA = double-stranded RNA.

Using a *Drosophila* double-stranded RNA genome-wide library (Heidelberg 2, BKN) (Horn *et al.*, 2010), we treated S2R+ cells with ∼21 000 double-stranded RNAs (∼98.8% coverage, covering ∼14 000 protein encoding genes and ∼1000 non-coding genes) for 48 h, followed by co-transfection of *K-renilla* and *L-firefly* mini-genes for a further 48 h. The ratio of K-renilla:L-firefly was then determined. We performed two replicates of screening and used criteria (K:L ratio ≥ 1.9 and Z-score > 1.5) to identify double-stranded RNAs that exhibited a similar or greater effect than double-stranded RNA *pasilla*. We identified 299 double-stranded RNAs (291 genes, ∼1.4% of the genome) which satisfied these criteria (Supplementary Table 2). Gene Ontology Annotation (Boyle *et al.*, 2004; Camon *et al.*, 2004) classifies these into 11 categories, including transcription/translation, post-transcriptional/post-translational modification, cell signalling, cell cycle, metabolism, oogenesis, cellular scaffolding and ion transportation (Fig. 2). Twenty-one per cent of the target gene products (i.e. proteins) are involved in post-transcriptional modification, including mRNA alternative splicing, polyadenylation and mRNA localization. This represents an enrichment compared to the genome, which contains 2.8% of genes involved in RNA processing (Fig. 2) (Boyle *et al.*, 2004). Notably, we identified *pasilla* validating our screen methodology. Furthermore, some transcripts, for example *Not1* (CG1884) and *crowded by cid* (CG5970), were hit twice by double-stranded RNAs (BKN20186 and BKN25930, BKN27434 and BKN46065, respectively) targeted to different regions.

### Behavioural screen to verify RNA interference targets influence seizure

The unidentified *bas^1^ Drosophila* mutation exhibits seizure-like behaviour when adult flies are exposed to strong sensory stimuli (e.g. vortexing) (Grigliatti *et al.*, 1973; Parker *et al.*, 2011*a*). As previously stated, manipulations that increase inclusion of exon K rescue seizure-like behaviour in bang-sensitive mutants (Lin *et al.*, 2012). To test whether knockdown of the 291 genes, identified in our double-stranded RNA screen, similarly rescue seizure in a bang-sensitive mutant, we performed a behavioural screen by expressing UAS-RNAi constructs in cholinergic neurons (the principle excitatory neurotransmitter of the insect CNS) in *bas^1^*.

**Figure 3:**
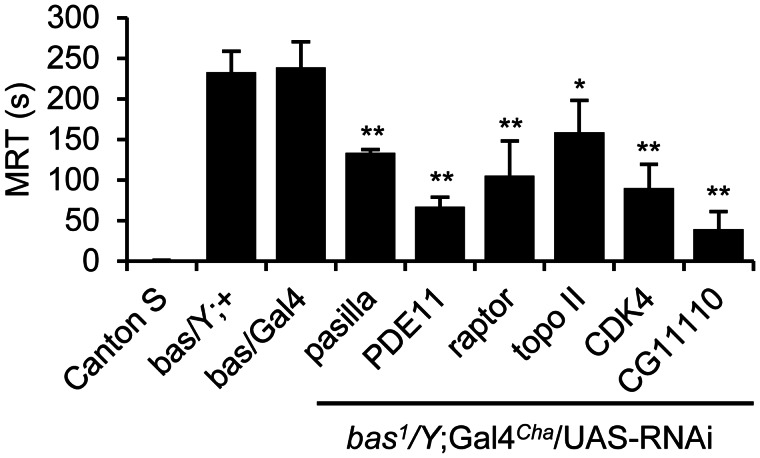
**UAS-RNAi lines rescue induced-seizure duration of *bas^1^* mutant flies**. Flies were subjected to a mechanical shock (10 s vortex) and the mean recovery time (MRT) was measured. *Bas^1^/Y* and *bas^1^/Y*;Gal4*^Cha^*/*+* (denoted *bas*/Gal4) male flies show similar mean recovery times (232.7 ± 26.2 and 238.8 ± 31.5 s, respectively). UAS-RNAi lines shown knockdown gene expression of *pasilla*, *phosphodiesterase 11* (*PDE11*), *raptor*, *topoisomerase II* (*topo II*), *cyclin-dependent kinase 4* (*CDK4*) and CG11110. All significantly reduced *bas^1^* seizure duration to 133.3 ± 4.4, 66.9 ± 11.2, 105.1 ± 43.2, 158.6 ± 39.6, 89.7 ± 29.3 and 39.2 ± 21.6 s, respectively. Values (mean ± SD for *n* = 5) were compared by ANOVA with Tukey’s post-test and results were deemed significant at *P ≤ 0.05 or **P ≤ 0.01.

We individually determined the mean recovery time of 265 RNAi candidates, which are currently available from the Vienna *Drosophila* Resource Centre. As expected, knockdown of *pasilla* significantly rescues seizure duration (133.3 ± 4.4 versus 238.8 ± 31.5 s; *bas^1^/Y*;Gal4*^Cha^*/UAS-RNAi *pasilla* versus *bas^1^/Y*;Gal4*^Cha^/+*, *P* ≤ 0.01, Fig. 3) mirroring its previously reported effect in *slamdance* (*sda*) (Lin *et al.*, 2012). Of the RNAi lines tested, 97 achieved significant behavioural rescue of seizure in *bas^1^* (Supplementary Table 3). Compared to RNAi *pasilla*, 45 (46%) lines show a statistically similar effect, while 52 (54%) exhibited a significantly stronger reduction of seizure duration. Amongst these genes are *Pde11* (CG15159), *raptor* (CG4320), *Topo II* (CG10223), *Cdk4* (CG5072) and a serine-type peptidase (CG11110) (Fig. 3). These genes are of particular interest because the protein products are already implicated in epilepsy and some are the focus of current clinical trials (Loscher *et al.*, 2013).

**Figure 4:**
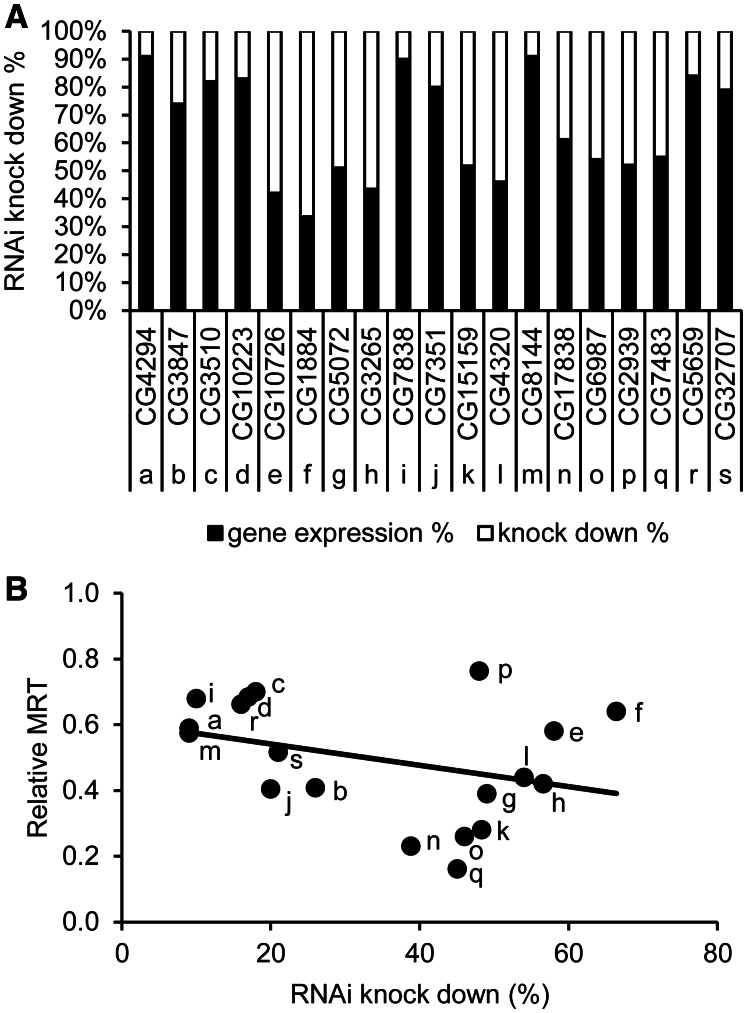
**RNAi-mediated knockdown of gene expression in the *bas^1^* mutant does not correlate to seizure reduction**. Male flies of 19 UAS-RNAi lines that spanned the effective range of seizure observed were crossed with *bas^1^*;Gal4*^Cha^* virgin females. The total RNA of F1 male fly heads (*bas^1^/Y*;Gal4*^Cha^*/UAS-RNAi) were extracted and quantitative RT-PCR performed to examine RNAi knockdown efficiency. (**A**) Black bars shows gene expression percentage, while the complementary white bars shows the RNAi knockdown percentage. RNAi knockdown efficiency ranges between 9 and 66%. The letters a–s and the corresponding CG numbers along the *x*-axis indicate the individual UAS-RNAi lines (see Supplementary Table 2 for the detail of these genes). (**B**) RNAi knockdown efficiency plotted against the relative mean recovery time (normalized to the controls *bas^1^/Y*;Gal4*^Cha^*/*+*) of each *bas^1^/Y*;Gal4*^Cha^*/UAS-RNAi line tested. The letters a-s indicate the corresponding CG numbers shown in (**A**). The line of best fit is not significantly different to a horizontal line (representing no correlation, ANOVA). MRT = mean recovery time.

To test whether the efficacy of seizure rescue is dictated by knockdown efficiency of the RNAi constructs, we selected 19 genes that spanned the effective range of seizure rescue observed: [CG1884 (*Not1*), CG2939 (*sloppy paired 2*), CG3265 (*Eb1*), CG3510 (*Cyclin B*), CG3847, CG4294, CG4320 (*raptor*), CG5072 (*Cdk4*), CG5659 (*ariadne 1*), CG6987 (*SF2*), CG7351 (*PCI domain-containing protein 2*), CG7483 (*eIF4AIII*), CG7838 (*Bub1-related kinase*), CG8144 (*pasilla*), CG10726 (*barren*), CG10223 (*Topo II*), CG15159 (*Pde11*), CG17838 (*Syncrip*) and CG32707 (*Anaphase Promoting Complex subunit 4*)]. We used quantitative RT-PCR to determine knockdown efficiency of each RNAi construct and plotted this against seizure rescue (Fig. 4). Knockdown efficiency ranges between 9 and 66% (Fig. 4A) but, importantly, does not significantly correlate to seizure reduction (line fit is not significantly different from a ‘zero’ horizontal line) (Fig. 4B). This suggests that seizure rescue is dependent on the targeted gene and not the efficiency of the RNAi construct.

**Figure 5:**
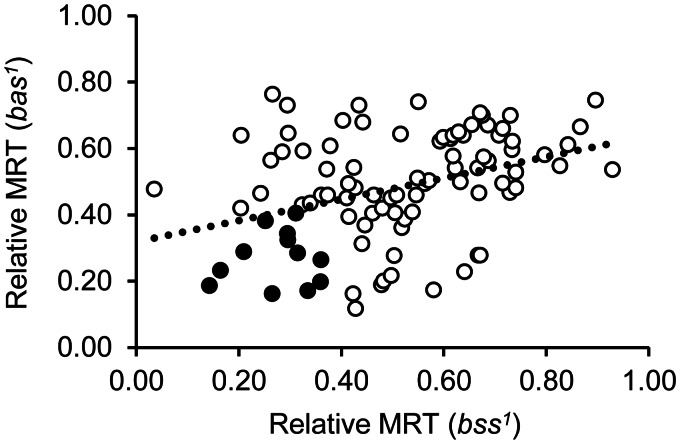
**UAS-RNAi lines that effectively reduced seizure duration in *bas^1^* are similarly effective in *bss^1^***. RNAi-mediated gene knockdown achieved by expressing UAS-RNAi using *Cha^B19^*-Gal4, in *bas^1^* and *bss^1^* mutant flies are compared. Flies of *bas^1^/Y*;Gal4*^Cha^*/UAS-RNAi or *bss^1^/Y*;Gal4*^Cha^*/UAS-RNAi were subjected to a mechanical shock (10 s vortex). Relative mean recovery time (MRT), normalized to control *bas^1^/Y*;Gal4*^Cha^*/*+* or *bss^1^/Y*;Gal4*^Cha^*/*+*, are plotted. Ninety-five (out of 97) of the UAS-RNAi lines significantly reduced seizure duration in both *bas^1^* and *bss^1^*. The line of best fit is significantly different from zero, showing a significant correlation (*P* ≤ 0.01, ANOVA). Twelve UAS-RNAi lines that show strong rescue effect are marked as solid circles. See Supplementary Table 3 for relative mean recovery time values.

To determine whether the rescue of seizure duration in *bas^1^* also occurs in other bang-sensitive mutants, we expressed 97 UAS-RNAi constructs, which significantly rescue seizure behaviour in *bas^1^*, in the alternate *bss^1^* mutation. This mutant carries a missense (hypomorphic) mutation of *DmNa_v_* and exhibits the most severe seizure-like phenotype of any bang-sensitive *Drosophila* mutant (Parker *et al.*, 2011*b*). Ninety-five RNAi lines, including RNAi *pasilla*, rescue seizure behaviour in *bss^1^* (Fig. 5). In general, RNAi lines that effectively rescued mean recovery time in *bas^1^* are similarly effective in *bss^1^*. The degree of seizure rescue observed in both genetic mutants (i.e. line of best fit) shows a relationship that is significantly different to zero at *P* ≤ 0.01 (zero representing a horizontal, no correlation, line) (Fig. 5). Of the 97 UAS-RNAis we tested, 95 lines (98%) significantly rescued mean recovery time (*P ≤ *0.05) in both *bas^1^* and *bss^1^* mutants. According to Gene Ontology annotation (Boyle *et al.*, 2004; Camon *et al.*, 2004), 20% of these 95 RNAis are classified into post-transcriptional modification category (Fig. 2). Twelve UAS-RNAi lines produced particularly strong rescue in both bang-sensitive mutants (>60% rescue, identified as solid circles in Fig. 5): the most effective amongst these were *Cell division cycle 5 ortholog* (CG6905), *Syncrip* (CG17838), CG5418 and *eIF4AIII* (CG7483) (Supplementary Table 3). Genes that, when knocked down, potently rescue seizure duration in both mutants are likely to work through well-conserved pathways and may, therefore, be optimal candidates to take forward to mammalian seizure studies.

### Rescue of seizure by small molecule inhibitors

The ability of known AEDs to suppress seizure in *Drosophila* provides further validation that this insect model is appropriate to identify and evaluate new anticonvulsant compounds (Reynolds *et al.*, 2004; Marley and Baines, 2011). Our double-stranded RNA screen has identified a number of genes, the protein products of which are already the subject of study for novel AED design. These include PDE11, RAPTOR (TOR-signalling), *TOPO II*, CDK4 and a serine-type peptidase (Zindy *et al.*, 1997; Song *et al.*, 2008; Lukasiuk *et al.*, 2011; Meng *et al.*, 2013; Nieoczym *et al.*, 2013).

**Figure 6:**
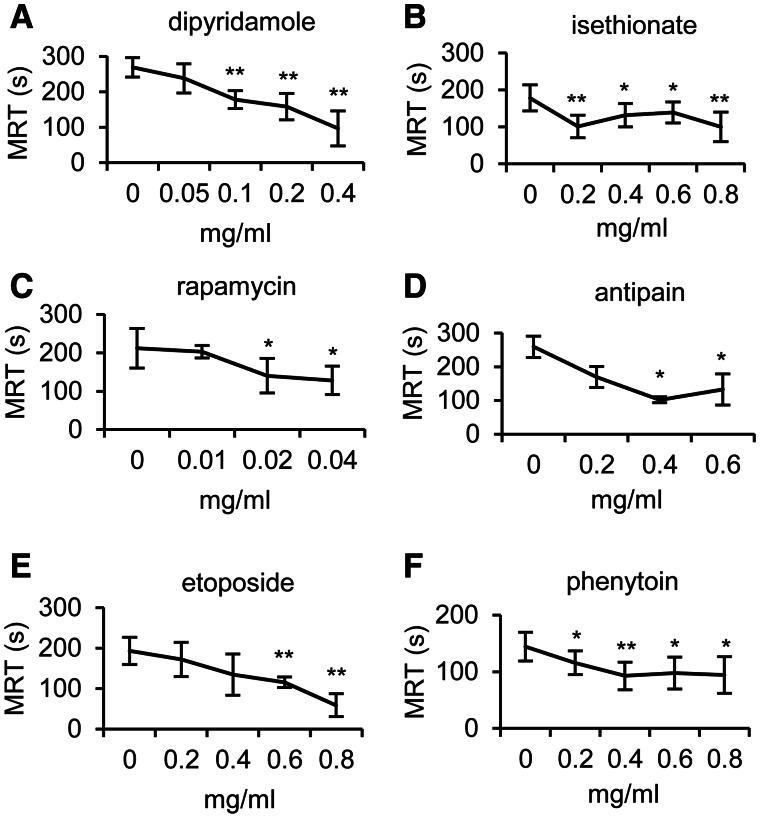
Small molecule inhibitors rescue seizure behaviour in *bas^1^* mutant flies. Acutely fed chemical inhibitors, (**A**) dipyridamole, (**B**) isethionate, (**C**) rapamycin, (**D**) antipain, (**E**) etoposide and (**F**) phenytoin (used as a positive control), to *bas^1^* adult flies for 24 h. Flies were then subjected to a mechanical shock (10 s vortex) and mean recovery time (MRT) calculated. Each drug exhibits a dose-dependent and significant reduction in seizure duration. Values (*n* = 5, mean ± SD) were compared by ANOVA with Tukey’s post-test and results were deemed significant at *P ≤ 0.05 or **P ≤ 0.01.

To further validate our screen, we determined if inhibition of these proteins is, as we might predict, anticonvulsive in *Drosophila*. To do so, we identified known chemical inhibitors and fed these to *bas^1^* mutant flies. Drugs used were dipyridamole (phosphodiesterase inhibitor), rapamycin (inhibit TOR-signalling), etoposide (Topo II inhibitor), isethionate (CDK4 inhibitor), and antipain (serine-type peptidase inhibitor). Exposure of adult *bas^1^* flies to these drugs, 24 h before testing, show that each is sufficient to produce a dose-dependent and significant reduction in seizure duration comparable to phenytoin, a potent anticonvulsant in both flies and mammals (Fig. 6). The amount of drug that each fly ingested was not measured and is, therefore, unknown. That these drugs, which target the protein products of the genes identified in our screen, are effective anticonvulsants not only validates our screen, but provides significant confidence that we have identified many additional, but as yet un-characterized proteins that may prove to be exploitable for novel AED design.

### Dipyridamole decreases I_NaP_ and exon L inclusion

**Figure 7:**
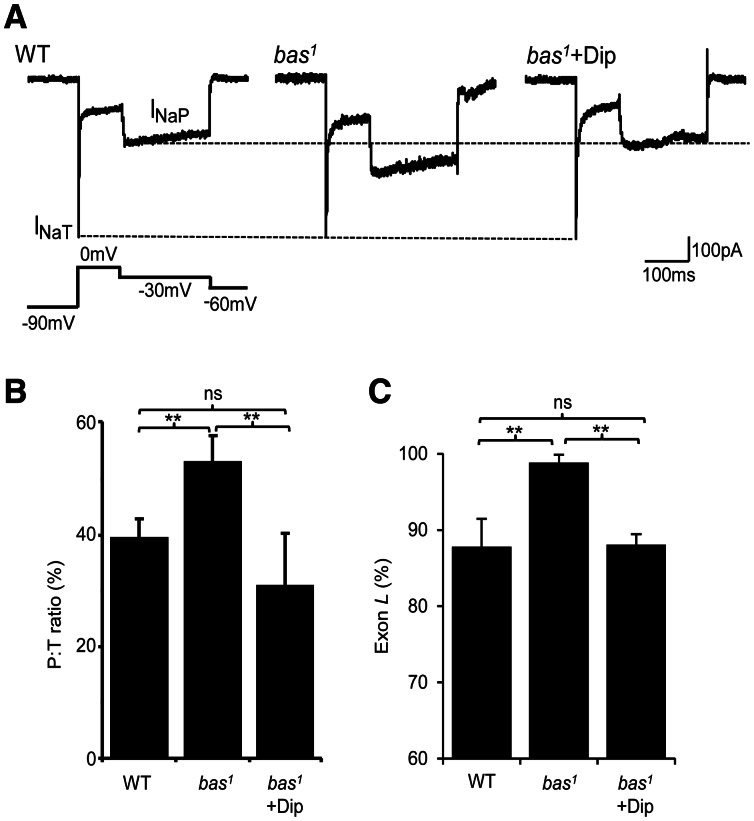
Dipyridamole decreases I_NaP_ and *DmNa_v_* exon L inclusion in the *bas^1^* mutant. (**A**) Whole-cell voltage-clamp recordings from third-instar anterior corner cell motoneurons show a marked increase of I_NaP_ in *bas^1^* compared with wild-type (WT), without effect to I_NaT_. Exposure to dipyridamole (Dip; 0.4 mg/ml) rescues the increase in *bas^1^*. *Inset* shows voltage protocol used to elicit Na^+^ currents (−90 mV/200 ms; 0 mV/100 ms; −30 mV/200 ms; −60 mV/100 ms). (**B**) Average values for the persistent to transient current (P:T) ratio for the three conditions shown in (**A**). P:T ratios are 39.4 ± 3.4, 53.1 ± 2.4 and 30.9 ± 9.2%, respectively, (*n* = 8). (**C**) Analysis of splicing of *DmNa_v_* in whole CNS shows that exon L inclusion in *bas^1^* is significantly increased compared to wild-type. Exposure of *bas^1^* to dipyridamole (0.4 mg/ml) is sufficient to decrease inclusion of exon L. Values are 87.8 ± 3.6, 98.9 ± 1.0 and 88.1 ± 1.4%, respectively, (*n* = 3). Pairwise comparisons were analysed for significance using a Student’s *t*-test at **P ≤ 0.01 and non-significant at *P* > 0.05 (ns).

The seizure phenotype characteristic of bang-sensitive mutants (i.e. *sda* and *eas*) is associated with increased inclusion of exon L in *DmNa_v_* and increased I_NaP_ in central motor neurons (Marley and Baines, 2011; Lin *et al.*, 2012; Lin and Baines, 2015). Similarly, *bas^1^* exhibits an increased I_NaP_ compared to wild-type. A persistent to transient current (P:T) ratio was measured by whole-cell voltage-clamp from the anterior corner cell (aCC) motorneuron (comparing I_NaT_ produced at 0 mV to I_NaP_ at −30 mV) in *bas^1^* and determined to be 53.1 ± 2.4% compared to 39.4 ± 3.4% in wild-type (*P* ≤ 0.01). Feeding dipyridamole (0.4 mg/ml) to *bas^1^* larvae significantly reduced the P:T ratio (30.9 ± 9.2%, *P* ≤ 0.01), through a specific reduction of I_NaP_ (Fig. 7A and B). Increased I_NaP_ expression correlates with increased exon L inclusion in *bas^1^* neurons (98.9 ± 1.0% versus 87.8 ± 3.6%, *bas^1^* versus wild-type, *P* ≤ 0.01). Exposure of *bas^1^* larvae to dipyridamole also rescued exon L inclusion to wild-type levels (88.1 ±1.4%, *P* ≤ 0.01, Fig. 7C). Thus, the anticonvulsive properties of dipyridamole are likely mediated through its ability to alter splicing of *DmNa_v_* to favour the K-exon variant that is associated with a smaller I_NaP_. We have yet to determine if the other small molecule inhibitors described above act in a similar manner but, based on the action of dipyridamole, there is every reason to predict that they will.

## Discussion

Despite an availability of numerous clinically-approved AEDs, 20–30% of epilepsy patients fail to respond to drug treatment (Sillanpaa and Schmidt, 2006; Loscher and Schmidt, 2011; Brodie *et al.*, 2012). Even for those patients that respond, debilitating side-effects can, and often do, arise. A common and effective target of many AEDs is the Na_v_ channel, but the inability of existing drugs to discriminate between reducing I_NaP_ without also affecting I_NaT_ limits their effectiveness. To date, no clinically-approved AED shows specificity for just I_NaP_. A recent study fully illustrates the efficacy of seizure rescue achievable by selective block of I_NaP_ (Anderson *et al.*, 2014) indicative that this target is likely to produce better, and perhaps more tolerable, AEDs. Taking advantage of our previous demonstration that splicing selectively regulates I_NaP_ in *DmNa_v_* (Lin *et al.*, 2009), we now identify 95 genes that, on knockdown, result in significant rescue of seizure duration presumably through potent reduction of I_NaP_. The protein products of these genes represent a valuable resource for the potential design of novel AEDs.

Of the 291 genes we identified, 13 belong to the *Cyclin/Cdk* family. Moreover, seven of the corresponding RNAi lines, *Cdk1* (CG5363), *Cdk2* (CG10498), *CDC45L* (CG3658), *Cdc5* (CG6905), *Cyclin B* (CG3510), *Cyclin D* (CG9096) and *Cdk4* (CG5072) significantly rescue seizure duration in both *bas^1^* and *bss^1^* mutants (Supplementary Table 3) indicative of common and exploitable mechanisms. We also show that acute feeding of isethionate, a CDK4 inhibitor, to *bas^1^* adult flies, rescues seizure duration. This over-representation implicates that cyclin/CDK function may be a tractable target for AED design. It is no surprise, therefore, that cyclin/CDKs have been implicated in epileptogenesis. For example, cyclin B1 upregulation is observed in the hippocampus of pentylenetetrazole (PTZ)-kindled rats (Pavlova *et al.*, 2006) and patients with temporal lobe epilepsy (Nagy and Esiri, 1998). Similarly, administration of kainite (KA) upregulates cyclin D1 expression in wild-type mice and loss of one copy of *cyclin D1* (*cyclin D1^+/^^−^* heterozygous mice) prevents kainite-induced seizure (Liu *et al.*, 1996; Timsit *et al.*, 1999; Koeller *et al.*, 2008). We also identified an unknown gene (CG31694), which regulates the JAK/STAT (Janus tyrosine kinase/signal transducer and activator of transcription) pathway (Muller *et al.*, 2005). The JAK/STAT pathway is upregulated in pilocarpine- or kainite-induced status epilepticus, which results in temporal lobe epilepsy in rodents (Choi *et al.*, 2003; Xu *et al.*, 2011). Administration of the JAK/STAT inhibitor, WP1066, reduces the severity of pilocarpine-induced seizure and downregulates downstream target transcripts of JAK/STAT, including *cyclin D1* (Grabenstatter *et al.*, 2014). Our findings raise the possibility that seizure induction results in activation of JAK/STAT signalling, through regulation of cyclin/CDK expression.

Our screen identifies many additional genes that may prove exploitable for novel AED development. Notable amongst these are *Pde11* (CG15159) and *raptor* (CG4320). Aberrant cAMP/cGMP levels are reported in human epilepsy and animal seizure models. For example, elevated cGMP and cAMP has been reported in the cerebral cortex, cerebellum and hippocampus following chemical-induced seizure (Ferrendelli *et al.*, 1980; Kohno *et al.*, 1997). Repeated injections of cAMP analogues into rat amygdala produced progressively more severe seizure behaviours similar to that induced by electrical kindling (Yokoyama *et al.*, 1989). The role of phosphodiesterase inhibitors for the treatment of seizure is more controversial. For example, sildenafil, a phosphodiesterase-5 inhibitor, shows anti-convulsant action in the mouse 6-Hz psychomotor seizure model (Nieoczym *et al.*, 2013) but exhibits pro-convulsant activity in PTZ-induced mouse clonic seizure model (Montaser-Kouhsari *et al.*, 2011). Inconsistency may derive from the expression of multiple phosphodiesterases in different brain regions (Domek-Lopacinska and Strosznajder, 2005), the ability of inhibitors to cross the blood–brain barrier (Liebenberg *et al.*, 2012) and/or the dose of proconvulsants used for seizure induction (Bankstahl *et al.*, 2012). In our screen, knock down of *Pde11* increases *DmNa_v_* exon *K* inclusion and UAS-RNAi*^Pde11^* expression rescues both *bas^1^* and *bss^1^* seizure duration. We also found that the phosphodiesterase inhibitor, dipyridamole, significantly reduced seizure duration. Dipyridamole produces a marked increase in the threshold for the onset of tonic extension in the PTZ-induced rodent seizure model (Akula *et al.*, 2008).

The direct interaction of raptor and mTOR is required for mTOR signalling (Hara *et al.*, 2002; Kim *et al.*, 2002). mTOR is a serine/threonine kinase involved in the highly conserved PI3K-Akt signalling pathway. It has recently been reported that hyperactivation of mTOR signalling is followed by seizure induction in rat and mouse models (Waltereit *et al.*, 2006; Grabenstatter *et al.*, 2014). Administration of mTOR inhibitors, i.e. rapamycin, prevents the development of absence seizure in WAG/Rij rats (Russo *et al.*, 2013), kindling seizure in *Tsc1*^GFAP^CKO mice (Zeng *et al.*, 2008) and kainite-induced status epilepticus in rats (Macias *et al.*, 2013). As such, the mTOR pathway has been identified as a ‘druggable’ target for the prevention of epileptogenesis (Lasarge and Danzer, 2014). In our screen, downregulation of *raptor* expression increased inclusion of *DmNa_v_* exon K and reduced seizure duration of both *bas^1^* and *bss^1^*. Furthermore, ingestion of rapamycin also effectively ameliorated *bas^1^* seizure duration.

Identifying seizure suppressor genes in *Drosophila* has proven effective for identifying mechanisms underlying seizure and identifying novel targets for AED design (Kuebler *et al.*, 2001; Hekmat-Scafe *et al.*, 2005; Parker *et al.*, 2011*a*). For example, *topoisomerase 1* (*top1^JS^*) and *gilgamesh* mutant flies, as well as the topoisomerase 1 inhibitor, camptothecin, reduce the severity of *bss^1^* seizure behaviour (Song *et al.*, 2007; Howlett *et al.*, 2013). In this study, the candidates of our screen are seizure suppressor genes which regulate a common downstream gene transcript, *DmNa_v_*. Knockdown of these genes is sufficient to rescue seizure behaviour of bang-sensitive mutants. However, the potential of the genes we identify here to become the basis for the design of novel AEDs goes beyond this study. The final choice will be dependent on many factors. These include how gene manipulation affects transcription/translation rates, in addition to splicing. Indeed, we see clear evidence for effects to transcription/translation of our mini-gene constructs but, importantly, identify many effective gene knockdowns that lack such an effect and only influence the splicing ratio to favour inclusion of exon K (Supplementary Table 2). We must also test for additional effects of gene knockdown *in vivo* including, but not limited to, effect to I_NaT_ and I_NaP_. *Na_v_* transcripts are heavily spliced and effects to other alternate exons and channel kinetics must be determined. Knockdown of *pasilla* affects splicing at *DmNa_v_* exons 12, 22, 23 in addition to 25 (Lin *et al.*, 2012). The change at exon 25 leads to increased inclusion of exon K which, in turn, reduces the amplitude of I_NaP_ without influence to I_NaT_ (Lin *et al.*, 2009, 2012). Finally, understanding which of the genes we identify show increased transcription following treatments to induce seizure, or in bang-sensitive mutant backgrounds, should also be informative. The expectation is that these genes are upregulated during/after seizure. Indeed, *Eb1* (CG4954), *shn* (CG7734) and *Relish* (CG11992), which we identify in our screen, are all upregulated in fly seizure mutants (Guan *et al.*, 2005). These follow-on studies, essential to narrow down our choice of genes to explore in detail, are readily achievable using *Drosophila*.

## Abbreviations

AED: antiepileptic drug
CDK: cyclin-dependent kinase
DmNa_v_: *Drosophila melanogaster* voltage-gated sodium channel
I_NaP_: voltage-gated persistent sodium current
I_NaT_: transient voltage-gated sodium current
JAK/STAT: Janus tyrosine kinase/signal transducer and activator of transcription
mTOR: mammalian target of rapamycin
Na_v_: voltage-gated sodium channel
